# Risk factors in the prediction of long‐term patency of Resonance metallic ureteric stent in malignant ureteric obstruction

**DOI:** 10.1002/bco2.14

**Published:** 2020-04-22

**Authors:** Brian S. H. Ho, Peter K. F. Chiu, Wayne Lam, Julius H. F. Wong, Charles K. W. Wong, Terence C. T. Lai, Chiu‐Fung Tsang, Ada T. L. Ng, Chi‐Kwok Chan, Wai‐Kit Ma, Chi‐Fai Ng, James H. L. Tsu

**Affiliations:** ^1^ Division of Urology Department of Surgery Queen Mary Hospital The University of Hong Kong Hong Kong Hong Kong; ^2^ Division of Urology Department of Surgery Prince of Wales Hospital Hong Kong Hong Kong; ^3^ SH Ho Urology Centre The Chinese University of Hong Kong Hong Kong Hong Kong

**Keywords:** malignant ureteric obstruction, metal stent, metallic ureteric stents, Resonance stent, ureteric obstruction

## Abstract

**Objectives:**

Cancer is the second leading cause of death globally in 2018 with an estimated 9.6 million deaths. The costs of managing malignant ureteric obstruction (MUO) is a significant burden to any healthcare system. However, the management of MUO has long been a challenge for urologists. The standard options of percutaneous nephrostomy or polymer double J stents are fraught with problems. We report a large patient series with long‐term follow‐up in the use of Resonance metallic ureteric stents to relieve MUO, and identification of risk factors associated with stent failure.

**Patients and methods:**

All patients with MUO who were arranged to have Resonance metallic ureteric stent insertion at two university hospitals were included in this cohort study, starting from June 2011 to July 2016. Data were retrieved retrospectively. The primary outcome was the total duration of stent patency before stent failure due to malignant disease progression. Stent failure was defined as ureteric obstruction identified on imaging (functional radioisotope scan or antegrade pyelogram), acute renal failure resolved by subsequent percutaneous nephrostomy, or any other cause requiring stent removal prematurely. Secondary outcomes were identification of factors associated with stent failure, grade III or above complication, and development of a risk‐adopted model to predict metallic ureteric stent patency rates in MUO patients. Median duration of functioning metallic ureteric stent was determined with Kaplan‐Meier survival curve.

**Results:**

A total of 124 renal units in 95 patients with MUO were eligible for the study, with a median follow‐up period of 22.9 months. About 106 (85.5%) renal units had successful metallic stent insertion, of whom 41 (33.1%) renal units ultimately progressed to ureteric obstruction despite the metallic stents, and required subsequent insertion of nephrostomies. Median duration of functioning metallic ureteric stents was 25 months. Female gender (HR 3.0, 95% CI: 1.3‐7.2, *P* = .014) and suspicious bladder lesion (HR 2.9, 95% CI: 1.4‐6.2, *P* = .005) were independent risk factors for stent failure, respectively. Stratifying patients into low (0 risk factor), intermediate (1 risk factor), and high (2 risk factors) risk groups, we found that this could predict the duration of stent patency in MUO with the metallic stents. (Low risk: 30.3 months vs intermediate group: 17.8 months vs high risk: 4.9 months, *P* < .001).

**Conclusion:**

Resonance metallic ureteral stents are able provide a median of 25 months of ureteric drainage in patients with MUO. Determining whether a patient has one or both risks factors (female gender and bladder lesion) will allow one to estimate the duration of metallic stent patency, which in turn may aid in determining cost‐effectiveness in individual patients.

## 1 INTRODUCTION

Cancer is the second leading cause of death globally in 2018 with an estimated 9.6 million deaths.^1^ The costs of managing malignant ureteric obstruction (MUO) is a significant burden to any healthcare system.^2^ However, the management of MUO has long been a challenge for urologists. Since definitive management of the underlying cause of obstruction may not always be feasible, the standard treatment of such patients include external drainage by insertion of percutaneous nephrostomies, or internal drainage with indwelling double J polymeric ureteric stents, commonly made of polyurethane, silicone or hydrogel material.

However, both options have their limitations. Nephrostomy tubes are susceptible to both blockage and dislodgement. They are unsightly and frequently cause discomfort to patients, which may reduce their quality of life. Polymer double J ureteric stents are also well‐known to cause discomfort and symptoms of bladder irritation. They require frequent exchanges every 3 to 6 months in order to reduce risk of device encrustation, biofilm development and associated urinary tract infections. However, internal double J stents still have 4.7% less febrile episodes when compared to percutaneous nephrostomy.^3^ Yet, the polymer nature of double J ureteric stents can be compressed by enlarging tumors resulting in stent failure. Thus, in recent years, there have been multiple attempts at developing stronger and more durable stents requiring less or no revisions.

The Cook Resonance metallic ureteric stents were first introduced in 2006 with a nonmagnetic nickel‐cobalt‐chromium‐molybdenum body in a spiral coil design. It has been demonstrated to be highly resistant to external compressive forces, being at least three to four times more robust than traditional polymer stents.^4^, ^5^ However, studies on the durability of Resonance metallic ureteric stents have been limited to small case series. This study aimed to identify risk factors associated with Resonance metallic ureteric stent failure due to malignant disease progression, and to propose a simple scoring system for the prediction of long‐term patency of metallic stent in patients with MUO.

## 2 PATIENTS AND METHODS

All patients with unilateral or bilateral MUO were offered Resonance metallic stent insertion at two university teaching hospitals between June 2011 to July 2016, except for patients who were clinically frail or patients who had previous urinary diversion surgery. Patients who were arranged for insertion of Resonance metallic ureteric stents were retrospectively identified using an institutional electronic patient records database. Patients with ureteric obstruction due to benign causes were excluded from analysis in this study. Patient demographics, level of ureteric obstruction, nature of obstruction, previous history of irradiation, presence of intravesical tumor or localized cystitis, need for intraoperative ureteric dilation, presence of preoperative double J stents or nephrostomy tube, preoperative and postoperative serum creatinine, and duration of functioning metallic ureteric stents were recorded. All the follow‐up appointments and imaging were arranged as necessary in the management of their cancer. There were no fixed follow‐up scheduled for this study besides the annual revision of Resonance metallic stents.

### 2.1 Insertion of Resonance metallic ureteric stents and subsequent management

All Resonance metallic ureteric stents were inserted in a standardized retrograde fashion under both cystoscopic and fluoroscopic guidance. Retrograde pyelogram was first carried out in all patients to confirm level of obstruction. Ureteric length was measured by catheterizing the ureter with an open ended ureteric catheter marked with a visual scale measurer. Gentle ureteric dilation (either by balloon or Teflon ureteric dilators) was performed at the discretion of the operating surgeon if required. Insertion of Resonance metallic ureteric stents were as per manufacturer's instructions. The position of the proximal coil of the metallic ureteric stents were confirmed with fluoroscopy and distal coil by cystoscopy. All metallic ureteric stents used were 6 Fr in size, with lengths measuring from 22 to 26 cm chosen according to ureteric length measured. All metallic ureteric stents were revised yearly as per manufacturer's recommendations.

### 2.2 Outcome measures

The primary outcome measure was the total duration of stent patency before stent failure due to malignant disease progression. Stent failure was defined as (a) ureteric obstruction identified on imaging, such as functional radioisotope scan or antegrade pyelogram, or (b) new onset acute renal failure resolved by percutaneous nephrostomy, or (c) any other cause requiring the removal of the metallic stent prematurely. Secondary outcome measures were identification of factors associated with failure of Resonance metallic ureteric stents and identification of grade III or above complication according to the Clavien‐Dindo classification.^6^

### 2.3 Statistical analysis

Data were analyzed using SPSS v20. Median duration of functioning metallic ureteric stent was determined with Kaplan‐Meier survival curve. Univariate and multivariate Cox regression analysis was used to identify risk factors. Fisher's exact test was used when analyzing other categorical variables. Ethics approval was obtained from the local institutional review board.

## 3 RESULTS

A total of 124 renal units in 95 patients with MUO were initially scheduled for metallic ureteric stent insertion from June 2011 to July 2016 for management of ureteric obstruction. Demographics of eligible patients are summarized in Table 1. Median time of follow‐up was 275 days (range 17 to 1990 days). One patient only had 17 days of follow‐up because she had died from her malignant disease. About 106 (85.5%) renal units had successful metallic ureteric stent insertion. Majority of the obstructions (64.5%) were limited to a single level; half of which occurred in the distal ureter (Table 1). Eighty one renal units (65.3%) had a polymer double J stent inserted previously, which were functioning in 78 renal units at the time of metallic ureteric stent insertion.

**TABLE 1:** Patient demographics

	No of renal units
Nature of obstruction	
Malignant	124 (100%)
Gynecological	46 (37.1%)
Cervical cancer	21
Uterine cancer	11
Ovarian cancer	14
Colorectal	34 (27.4%)
Colon cancer	10
Rectosigmoid cancer	23
Anal cancer	1
Genitourinary	18 (14.5%)
Prostate cancer	13
Bladder cancer	4
Renal cancer	1
Gastric cancer	11 (8.9%)
Breast cancer	6 (4.8%)
Hepatobiliary	4 (3.2%)
Pancreatic cancer	3
Liver cancer	1
Lung	2 (1.6%)
Squamous cell carcinoma of unknown primary	2 (1.6)
Lymphoma	1 (0.8%)
Gender	
Males	41 (33.1%)
Females	83 (66.9%)
Age	Median 62.1 years (range 31‐93 years old)
Median follow‐up duration	275 days or 9.0 months (range 17 to 1990 days)
Patient outcome	
Alive with disease	28 (22.6%)
Died from disease	95 (76.6%)
Died of unrelated cause	1 (0.8%)
Metallic ureteric stent insertion	
Successful	106 (85.5%)
Failed insertion	18 (14.5%)
Location of obstruction	
Single	80 (64.5%)
Upper ureter	25
Middle ureter	14
Lower ureter	41
Multiple levels	39 (31.5%)
Not specified	5 (4%)

**FIGURE 1:**
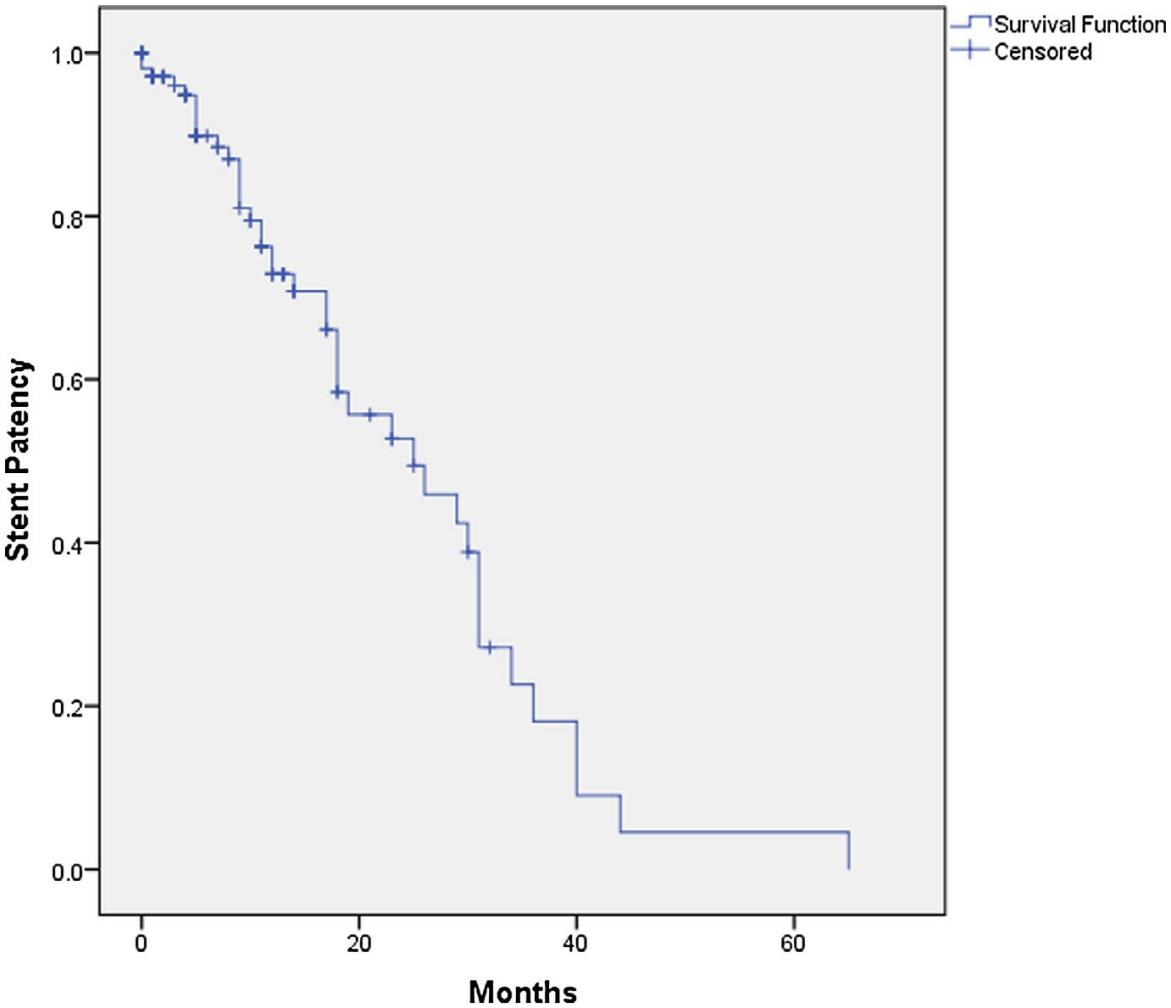
Duration of patent Resonance metallic ureteric stents in malignant ureteric obstruction

Of the 106 obstructed renal units that had successful Resonance metallic ureteric stent insertion, 41 (33.1%) renal units ultimately progressed to ureteric obstruction despite the metallic stents, and required subsequent insertion of nephrostomies. For the remaining 65 renal units, 50 renal units still had functioning metallic ureteric stents when the patient eventually passed away (95% of the deaths were due to the malignant disease causing the obstruction with the remaining 5% due to other medical causes) and in the last 15 renal units, the patients were still alive with functioning metallic stents. The crude mean duration of functioning metallic ureteric stents in MUO per renal unit was 268 days (8.3 months) from the day of insertion until either the stent failed or the patient passed away. But by using the Kaplan‐Meier survival analysis to censor a patient's death and annual revision of the metallic stents, the more accurate median duration of functioning metallic ureteric stent was 777 days (25 months) (Figure 1) before failure would occur due to disease progression. On Univariate analysis to identify risks factors for shorter stent patency in malignant causes of obstruction, female gender (*P* = .006), suspicious bladder lesion (*P* = .001), nonmetastatic disease (*P* = .049), and lack of adjuvant therapy (chemotherapy, radiotherapy or targeted therapy) (*P* = .040) were all associated with shorter functional duration of metallic stent (Table 2). Other factors such as age ≥ 60 years old, single vs multiple levels of obstruction, preoperative serum creatinine ≥ 150 µmol/L, different types of cancers, previous radiotherapy, presence of enlarged lymph nodes close to the ureter, use of ureteric dilators during insertion, mode of anesthesia and presence of double J stent were not predictive of stent failure. However, it must be noted that most of the renal units with prior of double J stent before the metallic stent insertion were functioning at the time of metallic stent insertion. Only three renal units had prior polymer double J stent insertion which failed, and then, went on to metallic Resonance stent insertion. Subsequent multivariate analysis with Cox regression showed that only female gender and suspicious bladder lesion were independent risk factors for stent failure, with hazard ratios 3.0 (95% CI: 1.3‐7.2, *P* = .014) and 2.9 (95% CI: 1.4‐6.2, *P* = .005), respectively.

**TABLE 2:** Risk factors for shorter metallic stent patency in malignant ureteric obstruction

	Univariate		Multivariate	
	Hazard ratio (95% CI)	*P* value	Hazard ratio (95% CI)	*P* value
**Female gender**	**3.2 (1.4‐7.3)**	**.006**	**3.0 (1.3‐7.2)**	**.014**
Age ≥ 60	1.4 (0.7‐2.5)	.352		
Preoperative serum creatinine ≥2 mg/dL (176.8 µmol/L)	1.6 (0.7‐3.8)	.246		
Previous radiotherapy to ureteric regions	1.1 (0.6‐2.0)	.765		
Previous double J stent inserted	1.0 (0.5‐2.1)	.935		
Ureteric dilation performed	1.4 (0.8‐2.7)	.273		
Local anesthesia	1.6 (0.7‐3.6)	.259		
Multiple level of obstruction	0.7 (0.3‐1.5)	.380		
**Suspicious bladder lesion (suggestive of bladder invasion or metastasis on cystoscopy)**	**3.1 (1.6‐6.1)**	**.001**	**2.9 (1.4‐6.2)**	**.005**
Colorectal cancer	0.9 (0.4‐1.7)	.683		
Genitourinary cancer	0.3 (0.1‐1.1)	.073		
Gynecological cancer	1.7 (0.9‐3.2)	.095		
Gastric cancer	0.4 (0.0‐21)	.323		
Enlarged lymph nodes close to ureter on imaging	1.1 (0.6‐2.2)	.679		
**Nonmetastatic disease (no lymph node or distant metastases)**	**1.9 (1.01‐3.6)**	**.046**	1.2 (0.6‐2.5)	.637
**No adjuvant therapy (chemotherapy or radiotherapy or targeted therapy)**	**1.9 (1.03‐3.7)**	**.040**	1.6 (0.8‐3.1)	.177
Postinsertion rise in serum creatinine	1.4 (0.7‐2.6)	.291		

**FIGURE 2:**
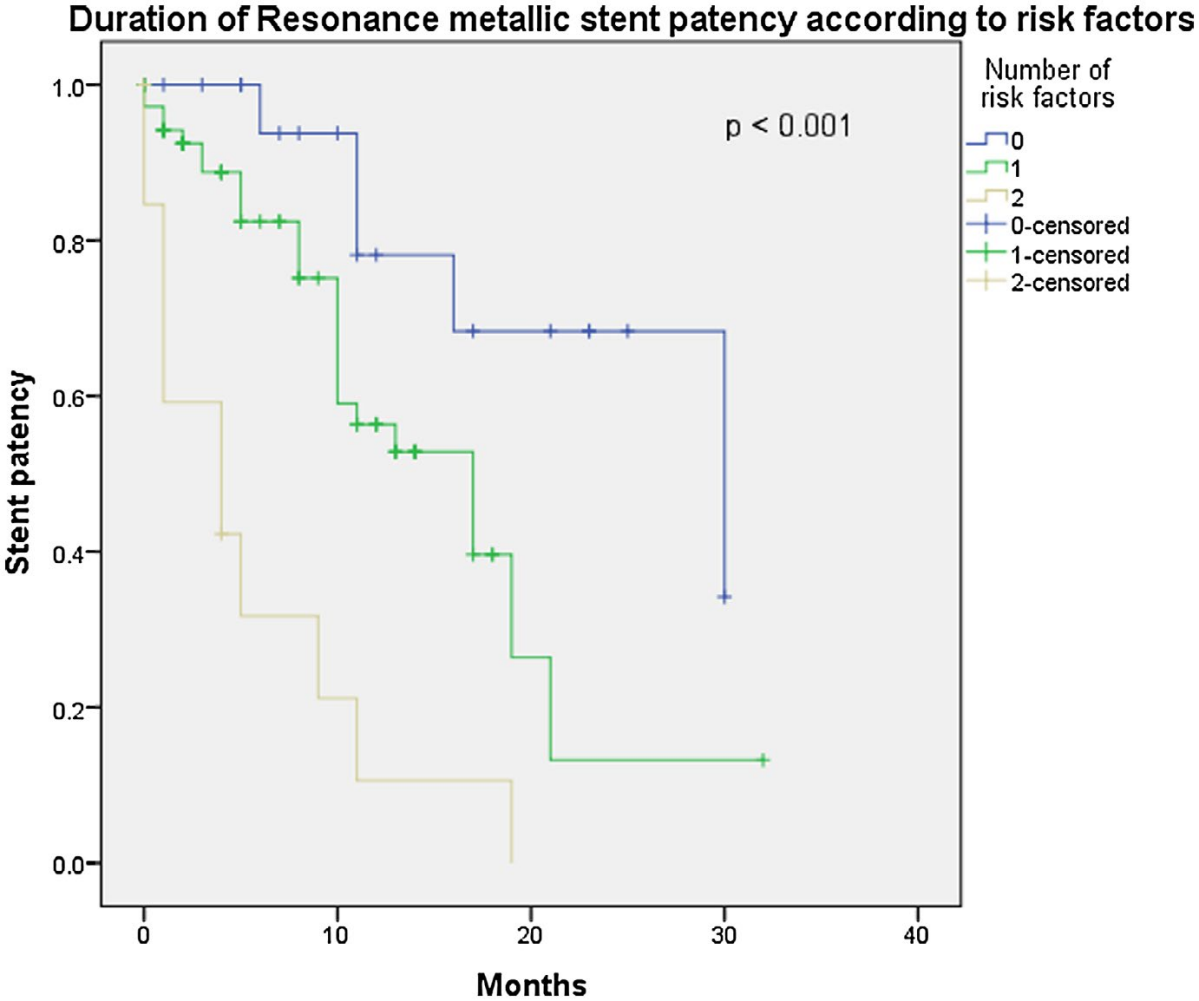
Resonance metallic ureteric stent patency in malignant ureteric obstruction when divided into 3 subgroups according to risk factors (female gender, bladder lesion) (*P* < .001)

On further analysis, we reclassified the patients according to a three‐tier system using the two risk factors of failure, namely, female gender and suspicious bladder lesion. Patients in the low‐risk group had zero risk factors, intermediate‐risk group had one risk factor, while those in the high‐risk group had two risk factors. We found that this could predict the duration of stent patency in MUO with the metallic stents in the low risk group functioning for a median of 925 days (30.3 months), 543 days (17.8 months) in the intermediate risk group, and 148 days (4.9 months) in the high‐risk group (*P* < .001) (Figure 2).

In terms of predictors of unsuccessful insertion metallic stent, only the absence of preoperative double J stent in situ was statistically significant (*P* < .001). The mode of anesthesia, whether general or spinal or local, did not have an impact on whether or not the metallic was successfully inserted. About 11 renal units in local anesthesia group had one failed metallic stent insertion, compared to 17 out of 113 renal units in the spinal or general anesthesia had failed stent insertion, which is statistically insignificant (*P* = .593).

### 3.1 Complications

None of the patients encountered grade 3 or 4 complications as a result of metallic ureteric stent insertion. However, one patient could not tolerate the subsequent storage bladder symptoms associated with the inserted metallic stent and required removal. One patient with local and systemic recurrence of rectal cancer after laparoscopic low anterior resection and adjuvant chemo‐irradiation had removal of the metallic stent as a result of the development of a vesicorectal fistula. Six renal units with metallic ureteric stents inserted were eventually found to have nonfunctioning kidneys after functional radio‐isotope scans at a later date.

## 4 DISCUSSION

The management of MUO has long been a challenge for urologists. Definitive management is usually limited due to the terminal or inoperable nature of disease causing obstruction. Both nephrostomy tubes and polymer double J stents have to be exchanged frequently and are associated with patient discomfort as well as reduced quality of life. Depending on the frequency of exchange and failure rates, the costs of managing MUO by polymer ureteric stent ranged from USD $4100 to $24 000 per patient per year.^2^, ^7^ Chung et al, in their study of 15 years of MUO patients who were treated with polymer ureteric stents, found a high stent failure rate of 42%.^8^ Thus, metallic ureteric stents were developed potentially to improve success rate in relieving patients with MUO without the need of a long‐term percutaneous nephrostomy tube.

Tightly coiled metallic ureteric stents (such as Memokath) and covered metallic ureteric stents (such as Allium ureteric stents) have been demonstrated to provide good outcomes. However, both techniques require ureters to be dilated to 14 F before they could be inserted, which may not be feasible in some malignant obstruction, and the dilation itself is associated with increased morbidity. Other options such as metallic mesh stents have also been used for MUO. However, they are plagued by tumor in‐growth and edema. Liatsiko et al conducted a study in which the patency rate of 27 metallic mesh stents was only 51.2% on follow‐up. All mesh stents were associated with early hyperplasia reaction and edema on antegrade nephrogram, which resulted in early obstruction in 14% of the mesh stents inserted. A high stent migration rate of 10.9% was also reported in their series.^9^ Considering these disadvantages of other metallic stents and the ease of insertion of Resonance metallic stents as well as their resistance against extrinsic compression,^5^, ^10^ Resonance stents were the preferred metallic stents at our units. However, these stents still need to be revised yearly.

In the literature, the reported duration of stent patency has been variable. Previous studies by Abbasi et al and Chow et al showed a mean duration of functioning metallic stent of 5.3 to 7.4 months.^11^, ^12^, ^13^, ^14^ Others reported the one year patency rates of Resonance metallic stents in malignancy ureteric obstruction ranged from 60% to 91%.^15^, ^16^, ^17^, ^18^

However, determining when the metallic Resonance stent will fail due to disease progression is better represented by Kaplan‐Meier survival since a significant portion of stents will be functioning at the time of the patient's demise, which would not be reflected in descriptive statistics. In our study, the gross median duration of functioning stents was 268 days (approximately 8.3 months), which is equivalent to Abbasi's findings. But if one uses the Kaplan‐Meier survival analysis instead, the metallic ureteric stents have a median duration of 777 days (approximately 25 months). This means that if patients survived long enough, they would be able to have two revisions of the metallic ureteric stents before stent failure occurred due to disease progression, or in other words equivalent to eight additional revisions of polymer double J stents (if revised at a 3 monthly interval) if the patient did not have the metallic stents inserted.

Different patients derive different durations of metallic ureteric stent patency, widely ranging from a month to few years before the stent can no longer drain the renal unit. Chow et al studied risk factors for Resonance metallic stent failure in 79 cancer patients and they found that serum creatinine of ≥2 mg/dL (176.8 µmol/L), age ≥ 60, and non‐lower gastrointestinal cancers were associated with shorter duration of functioning metallic stents.^13^ Wang et al have shown that previous radiation therapy had a lower patency rate of 50% compared to 92.3% in those patients without radiotherapy.^19^ Other studies have also analyzed risks factors of Resonance metallic stent failure and found that prostate cancer invasion into bladder, stone disease, ureteroileal anastomosis, bilateral obstruction, and previous failed mesh stents were all associated with shorter duration of function.^15^, ^20^, ^21^, ^22^ In our study, we have shown that only suspicious lesions in the bladder and female gender were independent risk factors for stent failure. Preoperative serum creatinine ≥ 2 mg/dL (176.8 µmol/L), different types of cancer, and previous radiation therapy were not associated with shorter duration of metallic stents patency in our study.

Since metallic double J stents rely on the patent distal loop to provide effective drainage of the upper tract, tumors involving the bladder (either by direct invasion or metastatic lesions) may grow and subsequently surround and encase the distal loop, resulting in the stent failure. Thus, it is logical to conclude that bladder lesions will result in shorter duration of patency for the metallic Resonance stents. This was also reported by Goldsmith et al. In their study of 37 stents in 25 patients with MUO, prostate cancer invasion into the bladder was associated with significantly increased risk of failure (HR 6.50, 95% CI 1.45‐29.20).^20^ It is interesting to note that female gender had higher failure rates, but when the data were further analyzed by cancer subgroups, with special attention to gynecological cancer or breast cancer, there were no significant difference found (Table 2). One possibility is that the female patients had more locally advanced cancers than the male counterparts in our series. We had tried to eliminate lymph node disease as well as distant metastases as confounding factors, but unfortunately the exact extent of the local staging at the time of the obstruction was usually unavailable. With a more locally advanced malignant disease, one would expect a higher likelihood of failure due to direct tumor infiltration into the ureter or just a greater compressive effect on the ureter by the mass.

Using the two independent risk factors for metallic stent failure, we can estimate the duration of Resonance metallic stent patency. The low‐risk group (no risk factors) would have a median functioning metallic stent duration of 925 days (30.3 months), intermediate‐risk group (one risk factor) 543 days (17.8 months), and high‐risk group (two risk factors) 148 days (4.9 months) (*P* < .001) (Figure 2).

The costs of managing MUO by polymer ureteric stent ranged from USD $4100 to $24 000 per patient per year.^2^, ^7^ Previous cost effective analysis by López‐Huertas et al comparing Resonance metallic stent vs polymer stent have shown that the metallic stent can provide a 43% cost reduction within a 12 month duration.^7^ Yuen et al's study in a public hospital in Hong Kong estimated the cost of each polymer insertion to be equivalent to USD $1437 and USD $5638 for each Resonance stent insertion.^23^ Using these numbers, four polymer stent exchanges (if one used the 3 monthly stents) would outweigh the cost of a Resonance metallic stent insertion. Thus, the high risk group would result in a financial disadvantage if Resonance metallic stent was inserted.

Although none of our patients experience Grade 3 or 4 complications as a result of the metallic stent insertions, there were six renal units (5.6%) that were found to be nonfunctioning on subsequent radioisotope scans (DTPA, MAG3). Most patients were followed up with regular renal function tests and imaging of the kidneys (ultrasound, computed tomography or magnetic resonance imaging) as were required by the management of their malignancies. However, serum creatinine is not sensitive for individual kidney function and assessment of hydronephrosis on imaging is very subjective, especially if it is across imaging modalities. The best method to detect obstruction would be to perform functional radioisotope scan regularly for all patients with metallic stents inserted but this would increase the radiation exposure for these patients as well as costs to the health care system. The ideal follow‐up protocol for metallic stents has yet to be elucidated.

We acknowledge that there are various limitations associated with this study. First, the patients included in the study did not have a strict standardized follow‐up protocol. Failure of the metallic ureteric stents may have occurred before the patient returned for follow‐up. Second, the precise TNM staging of the cancers were not known so the exact stage of the malignancy cannot be determined. Different patients with the same malignancy may be at different stages of the disease, which may have an impact on the severity and progression of their MUO. However, we tried to circumvent this problem by using the presence of metastasis and the presence of para‐aortic & iliac lymph nodes to help stratify the general severity of disease. Third, the cost benefits of Resonance vs polymer stents can vary significantly between countries due to differences in regional costs of hospitalization and clinical staff. However, with the three tier scoring system, one would be able to assess which patients it would be cost effective to insert Resonance metallic stents if they knew the exact cost of each polymer and Resonance stent insertion in their locality. Fourth, the proposed scoring system still need to be validated since the current study does not have enough patient number to perform validation. Lastly, we did not include patients with ureteroenteric reconstruction. Previously, Garg et al have stated in their study that such patients usually have high rates of distal migration with a mean time to stent migration of only 21 days. In fact, only one patient did not have any migration in their study.^24^ Thus, we did not offer Resonance stents for this cohort of patients.

## 5 CONCLUSION

The Resonance metallic ureteric stents has a good median functioning duration of 25 months for patients with MUO. Female gender and suspicious lesions in the bladder were independent risk factors for stent failure. The proposed three‐tier scoring system helps to predict long‐term Resonance metallic stent patency rates, which in turn may aid in determining cost‐effectiveness in individual patients.

## DISCLOSURE

None of the authors have any conflict of interests to disclose.
